# Supplementary material for: Population fluctuations and synanthropy explain transmission risk in rodent-borne zoonoses
Source: Nat Commun. 2022 Dec 7;13:7532. doi: 10.1038/s41467-022-35273-7 (PMC9729607; doi:10.1038/s41467-022-35273-7)
Supplement: Supplementary file 1 — Supplementary Information File [file 41467_2022_35273_MOESM1_ESM.pdf]

Population fluctuations and synanthropy explain transmission risk in rodent-borne zoonoses

Frauke Ecke[1, 2*], Barbara A. Han[3], Birger Hörnfeldt[1], Hussein Khalil[1], Magnus Magnusson[1, 4], Navinder J. Singh[1] and Richard S. Ostfeld[3]

[1] Department of Wildlife, Fish, and Environmental Studies, Swedish University of Agricultural Sciences, SE-901 83 Umeå, Sweden

[2] Organismal and Evolutionary Biology Research Programme, Faculty of Biological and Environmental Sciences, PO Box 65, FIN-00014 University of Helsinki, Finland

[3] Cary Institute of Ecosystem Studies, Millbrook, New York 12545, USA

[4] Swedish Forest Agency, Box 284, SE-901 06 Umeå, Sweden

* Corresponding author, email: Frauke.Ecke@helsinki.fi

Supplementary Figures 1-3

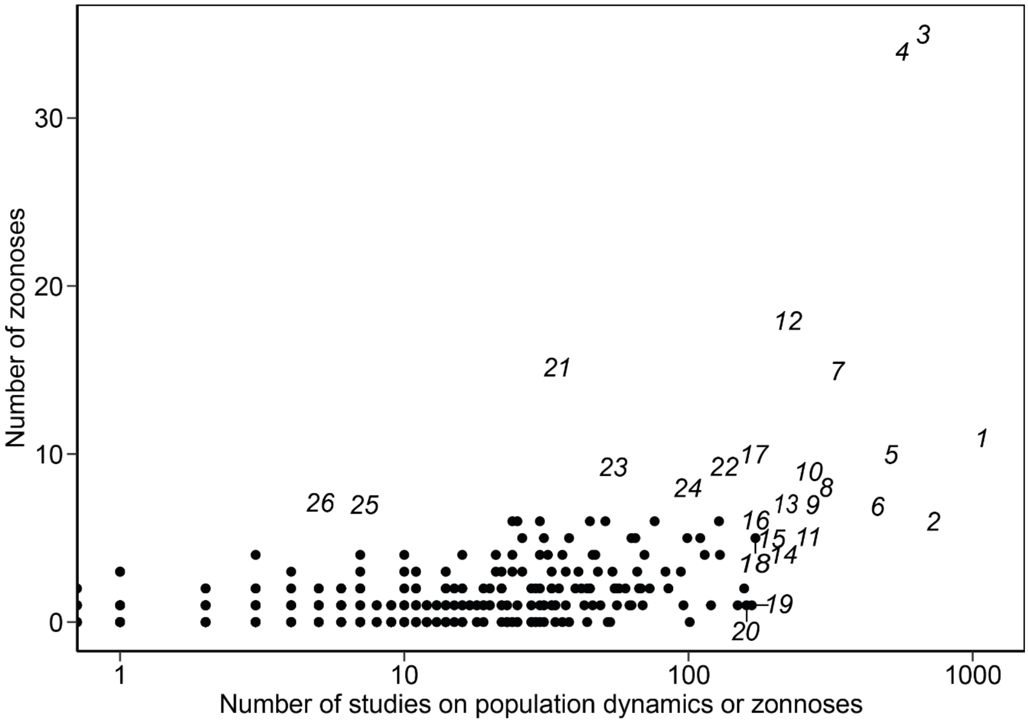

1 - *Mus musculus (AfAsAuEuNaSa)*
2 - *Myodes glareolus (AsEu)*
3 - *Rattus norvegicus (AfAsAuEuNaSa)*
4 - *Rattus rattus (AfAsAuEuNaSa)*
5 - *Peromyscus maniculatus (Na)*
6 - *Peromyscus leucopus (Na)*
7 - *Apodemus sylvaticus (AfEu)*
8 - *Microtus arvalis (AsEu)*
9 - *Microtus agrestis (AsEu)*
10 - *Apodemus flavicollis (AsEu*
11 - *Arvicola amphibius (AsEu)*
12 - *Apodemus agrarius (AsEu)*
13 - *Cynomys ludovicianus (Na)*
14 - *Mesocricetus auratus (As)*
15 - *Mastomys natalensis (Af)*
16 - *Castor canadensis (AsEuNaSa)*
17 - *Sigmodon hispidus (Na)*
18 - *Tamiasciurus hudsonicus (Na)*
19 - *Myodes gapperi (Na)*
20 - *Meriones unguiculatus (AsEu)*
21 - *Bandicota indica (As)*
22 - *Sciurus vulgaris (AsEu)*
23 - *Rattus exulans (AsAu)*
24 - *Arvicanthis niloticus (Af)*
25 - *Rattus tiomanicus (As)*
26 - *Bandicota savilei (As)*

Supplementary Figure 1. Relationship between the number of identified studies on population dynamics or zoonoses per rodent species and the number of zoonoses hosted by a rodent species (Spearman's rank correlation coefficient $r_{s,436}$ = 0.42, p = 0.000). For the 20 most-studied species, points have been replaced by labels (numbers centralized on points) for the respective species except for species 18-20 due to visibility. In addition, labels are shown for rodents that host more than five zoonoses irrespective number of studies. Continents where the species occur are given in parentheses (Af Africa, As Asia, Au Australia and Oceania, Eu Europe, Na North America, Sa South America).

Rodents exclusively occurring in Africa and Asia are generally less studied than species that exclusively occur in North America (See Supplementary Figure 2). However, the Asian reservoir Golden Hamster (*Mesocricetus auratus*) and the African reservoir Natal Mastomys (*Mastomys natalensis*) are the 14th and 15th most studied species, and the African species African Grass Rat (*Arvicanthis niloticus*) as well as the Asian species Greater Bandicoot Rat (*Bandicota indica*), Malaysian Field Rat (*Rattus tiomanicus*) and Savile's Bandicoot Rat (*Bandicota savilei*) are four of 18 species hosting at least seven zoonoses.

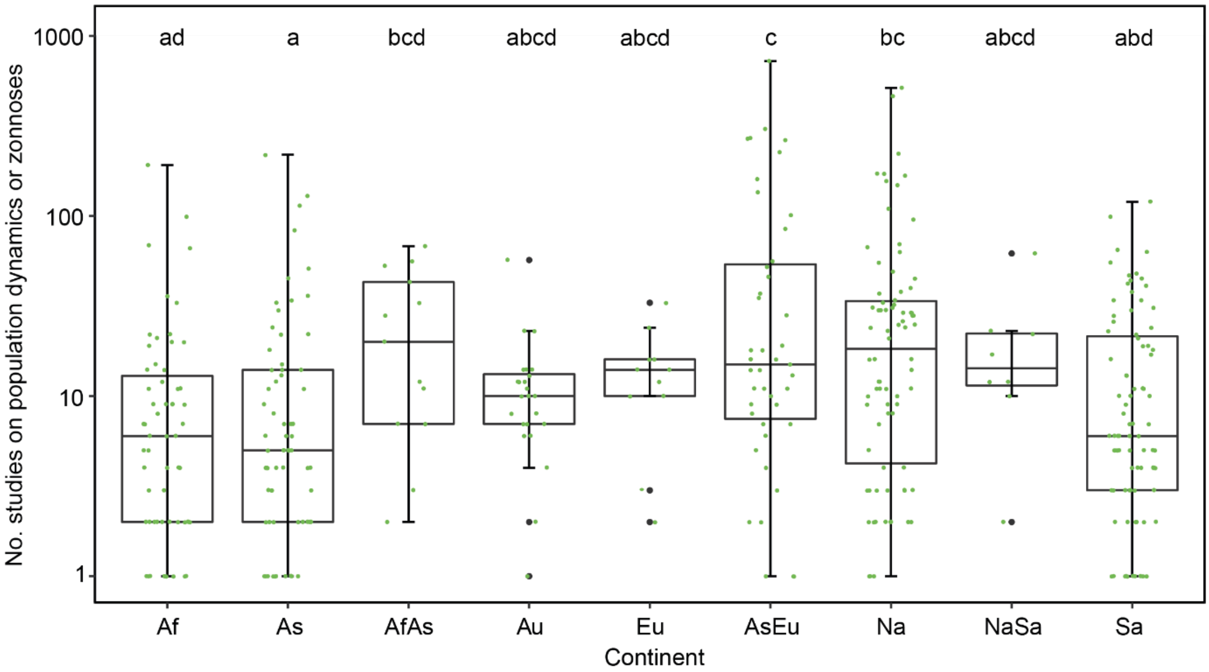

Supplementary Figure 2. Number of studies per rodent species on population dynamics or zoonoses per continent (Af Africa [*n* = 59], As Asia [*n* = 79], Au Australia and Oceania [*n* = 24], Eu Europe [*n* = 11], Na North America [*n* = 86], Sa South America [*n* = 90]). The groups AfAs (*n* = 13), AsEu (*n* = 44) and NaSa (*n* = 9) represent species that occur in both Africa and Asia, Asia and Europe and North and South America, respectively. Medians are given with boxes representing the 25th and 75th percentiles and whiskers 1.5 × the inter-quartile ranges. Only groups of continents with at least five species are shown. Each green dot represents a rodent species. Outliers are indicated by black dots. Different letters above plots indicate significant ($p<0.05$) differences among groups as tested by Kruskal-Wallis test with Dunn test for posthoc comparisons.

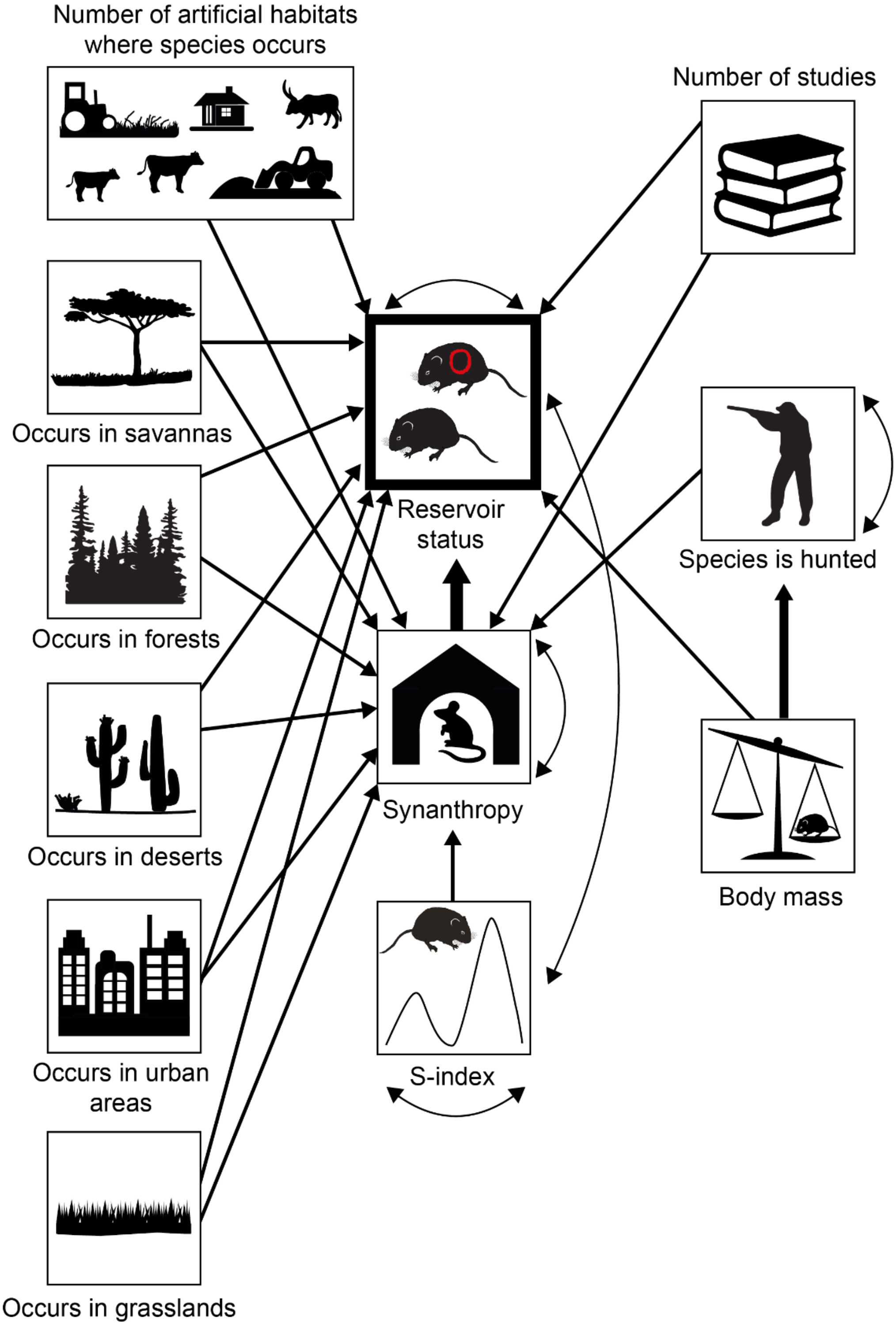

Supplementary Figure 3. The full (*a priori*) structural equation model linking reservoir status of rodent species (n = 269) with their synanthropy and hunting status, population fluctuations (s-index, log-transformed), and adult body mass, controlling for their occurrence in a range of habitats. One-sided (directional) arrows represent a causal influence originating from the variable at the base of the arrow, with the width of the arrow representing the standardised strength of the relationship. The final model presented in the main article excludes the non-significant pathways proposed here (see Figure 2 and Results section).
