# Supplementary material for: Population fluctuations and synanthropy explain transmission risk in rodent-borne zoonoses
Source: Nat Commun. 2022 Dec 7;13:7532. doi: 10.1038/s41467-022-35273-7 (PMC9729607; doi:10.1038/s41467-022-35273-7)
Supplement: Supplementary file 3 — Description of Additional Supplementary Files [file 41467_2022_35273_MOESM3_ESM.docx]

**Description of Additional Supplementary Files**

File Name: Supplementary Data 1

Description: Reservoir status, distribution range, life history traits, synanthropy, habitat preferences, hunting status, population fluctuations, references, and reservoir-disease associations per rodent species.

File Name: Supplementary Data 2

Description: Transmission modes, pathogen type, and references per rodent-borne zoonosis.
